# Chronic Kidney Disease After Lung Transplantation in Spain: A Retrospective Single-Center Analysis

**DOI:** 10.3390/jcm14072241

**Published:** 2025-03-25

**Authors:** Maria Luisa Serrano Salazar, Carlos Almonacid, Maria Marques Vidas, Paula López-Sánchez, Beatriz Sánchez Sobrino, Myriam Aguilar, Lucia Rubio Arboli, Eduardo Martínez Morales, Ana Huerta, Maria Valdenebro Recio, Piedad Ussetti, Jose Portoles

**Affiliations:** 1 Nephrology Department, Hospital Universitario Puerta de Hierro Majadahonda, Instituto de Investigacion Puerta de Hierro-Majadahonda-Segovia Arana, 28222 Madrid, Spain; mserranosalazar@gmail.com (M.L.S.S.); nefro_metodologia@yahoo.com (P.L.-S.); bssobrino@salud.madrid.org (B.S.S.); eduardo.mm1996@gmail.com (E.M.M.); ana.huerta@me.com (A.H.); mdev183@hotmail.com (M.V.R.); josem.portoles@salud.madrid.org (J.P.); 2 Pulmonology Department, Hospital Universitario Puerta de Hierro Majadahonda, IDIPHISA, 28222 Madrid, Spain; carlos.almonacid@salud.madrid.org (C.A.); myriamaguipe@yahoo.es (M.A.); pied2152@separ.es (P.U.); 3 Medicine Department, Facultad de Medicina, Universidad Autónoma de Madrid, IDIPHISA, 28029 Madrid, Spain; luciarubioarboli@hotmail.com

**Keywords:** lung-transplant, chronic kidney disease, estimated glomerular filtrate rate, renal replacement therapy

## Abstract

**Objectives**: Chronic kidney disease (CKD) among lung transplant (LTx) recipients has increased in recent decades. However, there is insufficient evidence regarding clinical outcomes, and current guidelines lack specific recommendations for its management. **Methods**: This single-center retrospective study included all patients who underwent LTx and were subsequently referred to a dedicated nephrology outpatient clinic. Major adverse renal events were defined as a composite event. **Results**: Eighty LTx recipients with underlying lung disease etiology such as cystic fibrosis, chronic obstructive pulmonary disease, or interstitial lung disease were included. The mean time from LTx to first nephrologist evaluation was 4.7 years with an eGFR of 31.7 mL/min/1.73 m^2^. LTx recipients experienced a 48% reduction in eGFR within the first few months after LTx. Rapid progressors require renal replacement therapy earlier than the slow progressors. Patients requiring dialysis had higher all-cause mortality compared to those who did not require dialysis. **Conclusions**: Early post-LTx functional impairment appears to be the most significant predictor for CKD progression and the eventual need for RRT. Although CNI toxicity is the most common cause of CKD, early nephrology evaluation can uncover other causes and promote early renoprotective measures. For this patient population, specific guidelines addressing CKD after LTx and a multidisciplinary approach are essential.

## 1. Introduction

Each year, between 350 and 400 patients undergo lung transplantation (LTx) in Spain. According to data from the Spanish National Organization Transplant (ONT) [1], the median survival of these patients is 6.4 years. Spain’s national public health system coordinates the entire process, from lung donation and transplantation to follow-up, through a network of seven public centers serving a population of 48 million, overseen by the ONT. These seven reference hospitals also operate kidney transplant and other solid organ transplant (SOT) programs, including liver and heart transplants. Additionally, 35 other centers host kidney transplant programs, with many more serving as organ procurement facilities.

The incidence of chronic kidney disease (CKD) among SOT recipients has risen significantly in recent years, becoming an increasingly common issue in our clinical practice [2,3]. Specifically, in LTx recipients, advancements in graft preservation, surgical techniques and immunosuppression therapies, such as calcineurin inhibitors (CNIs) and other drugs, have contributed to improved transplant success rates and prolonged patient survival. 

CNI toxicity is well-documented in kidney transplant patients but also in patients with hematopoietic cell and non-kidney solid organ transplants [4,5]. CNI toxicity can lead to acute kidney injury (AKI), and it is usually related to high drug serum concentrations, and, after its adjustment, it is often reversible. This acute injury appears to result from endothelial dysfunction, where a misbalance of an increase in vasoconstrictors and a decline in vasodilator substances leads to arteriolar vasoconstriction in the glomerulus, ultimately reducing the glomerular filtration rate [6]. Additionally, chronic CNI exposure contributes to CKD due to glomerular and vascular damage seen in kidney biopsies associated with tubular dysfunction and high blood pressure. While the underlying mechanism remains unclear, initial arteriolar damage seems to trigger widespread glomerulus and tubule–interstitial damage. Otherwise, other CNI renal effects include tubular dysfunction and, in rare cases, thrombotic microangiopathy (TMA), which can lead to severe AKI [7].

Moreover, expanded donor selection criteria have enabled transplantation in a broader patient population despite the presence of additional comorbidities. However, despite these advances, there is a lack of recent evidence on the prevalence, causes, progression, and outcomes of CKD in LTx recipients. Furthermore, specific recommendations for managing CKD in LTx are absent from both nephrologist-CKD guidelines [8] and heart and LTx guidelines [9]. 

In this context, we conducted a retrospective single-center study of our experience with LTx patients referred to our specialized nephrology outpatient clinic for SOT-related CKD. The objectives of this study were to describe the progression of renal function since LTx, identifying risk factors for worse CKD prognosis and an early need for renal replacement therapy (RRT) and identifying opportunities to implement nephroprotective measures. This study aims to raise awareness and facilitate the identification of areas for improvement and potential intervention strategies. 

Our goal is to characterize LTx recipients who develop CKD, not only due to CNI toxicity, and assess their outcomes while identifying risk factors for CKD progression.

## 2. Materials and Methods

Retrospective, single-center, observational study of patients who underwent LTx and were subsequently referred to the SOT-CKD clinic at the Hospital Universitario Puerta de Hierro in Majadahonda, Community of Madrid, Spain. This clinic is led by a nephrologist specializing in transplantation, based on clinical criteria established by pulmonologists. The study period covered patients from 2007 to 2020. All patients who attended at least one visit to our SOT-CKD clinic were included. 

A nephrologist collected clinical data from a shared electronic medical record used across all hospital departments, minimizing selection bias due to outcomes or missing data (including hospital admissions, complications, laboratory values, and clinical events). Data were collected in a custom-designed database (Microsoft Excel 2007^®^).

Demographic, clinical, and laboratory data, including serum creatinine and estimated glomerular filtration rate (eGFR) calculated using the CKD-EPI formula, were collected up to the present. Additionally, clinical events such as infections, pulmonary rejection episodes, and acute kidney failure (AKI), as well as details of immunosuppressive therapy, were documented. 

Major adverse renal events (MAREs) were defined as a composite outcome, including doubling serum creatinine and initiation of RRT or CKD-related mortality [10]. First-year CKD progression was defined as the percentage reduction in eGFR compared to pre-transplant values. Patients were classified as rapid progressors if they fell into the third tertile and as slow progressors if they fell into the first tertile.

Albuminuria was defined based on the urine albumin/creatinine ratio (UACR) and classified as A1, A2, or A3, depending on severity (ACR < 30, 30–300, or >300 mg/g, respectively). Leukocyturia was defined as five or more leukocytes per field, while microhematuria was diagnosed as the presence of three or more red blood cells per high-power microscopic field in urine sediment.

For kidney biopsies, optical microscopy was performed using standard stains (hematoxylin and eosin, PAS, methenamine silver, and Masson’s trichrome technique). Immunofluorescence study and electron microscopy were conducted when necessary for diagnosis.

Statistical analysis: Continuous variables were presented as means with standard deviation (SD) or median and interquartile range (IQR), while categorical variables were expressed as percentages. Group comparisons were conducted using a chi-square or Fisher’s exact test for qualitative variables and Student’s *t*-test/ANOVA or Mann–Whitney/Kruskal–Wallis test for quantitative variables. Normal distribution of data was assessed using the Kolmogorov–Smirnov test.

To analyze factors associated with combined MARE, univariate logistic regression was initially performed to estimate the odds ratio for each factor. A backward multivariate model was then applied, incorporating all variables with a *p*-value of <0.10, along with clinically relevant variables (such as CV/DM, due to their role as well-known risk factors for CKD progression), which were analyzed to select the most accurate model based on the R^2^ value. The probability of including or excluding a covariate was set at *p* (in) < 0.05 and *p* (out) > 0.1, respectively.

For the analysis of factors associated with CKD progression or the need for RRT, Fine and Gray’s proportional subdistribution hazards model or a Cox regression model (assuming that the censored data are not informative) were used. The necessity of an analysis of competitive events was evaluated using the Pepe Mori test. Variables were first assessed in a univariate model, followed by a backward multivariate model that included all variables resulting in a *p*-value of <0.10 in the univariate model, along with all clinically relevant variables (associated with CKD progression or need of RRT). The probability of including or excluding a covariate was set at *p* (in) < 0.05 and *p* (out) > 0.1, respectively.

A *p*-value < 0.05 was considered statistically significant. Statistical analysis was conducted using STATA 14.0 (Stata Statistical Software: Release 14. College Station, TX, USA: Stata Corp LP).

The study protocol was approved by the Ethics Committee of Hospital Universitario Puerta de Hierro (PI 64/24; 1 March 2024) and has been reported following the STROBE guidelines.

## 3. Results

**Figure 1:**
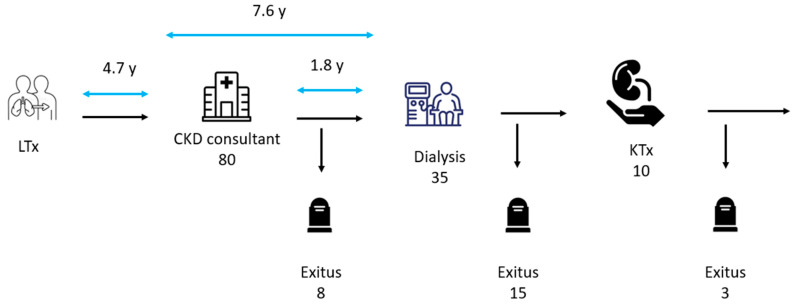
Flowchart outcomes of patients attended in our solid organ transplantation outpatient clinic. LTx: lung transplant, KTx: kidney transplant, CKD: chronic kidney disease; y: years.

Eighty patients who underwent LTx were referred to our SOT-CKD clinic and enrolled in the study (Figure 1).

The majority of patients had undergone bipulmonary transplantation (81%). The median age at the time of LTx was 49.7 years (SD 15.9), with males accounting for 56.3% of the cohort. The etiology of lung disease was as follows: chronic obstructive pulmonary disease (COPD) 33.8%, interstitial lung disease (ILD) 32.5%, cystic fibrosis (CF) 28.8%, and others 5%. At the time of LTx surgery, the median eGFR was 98.0 mL/min/1.73 m^2^, with an interquartile range (IQR) of [88.3–113.1]. None of the patients had pre-existing CKD. A total of 71.2% were treated with tacrolimus, while the remaining 28.8% received cyclosporine (28.8%). 

Referral to the SOT-CKD clinic occurred after a median duration of 4.7 years [IQR 2.7–8.2], with a median eGFR of 31.7 mL/min/1.73 m^2^ (median of 29 mL/min [23–37]), according to clinical criteria. The time from LTx to nephrologist referral was 5.8 years longer in the cystic fibrosis group compared to COPD patients (5.8 [3.4–8.2]; *p* < 0.001) and 4.5 years longer than those with diffuse interstitial lung disease (4.5 [2.1–6.9]; *p* < 0.001). As expected, cystic fibrosis patients were younger and had a lower prevalence of active smokers but a higher prevalence of diabetes mellitus (DM). It is noteworthy that the cystic fibrosis group had a higher incidence of undergoing lung re-transplantation.

At their initial nephrologist visit, patients presented comorbidities such as hypertension (80%), DM (53.8%), dyslipidemia (46.3%), and a history of previous cardiovascular events (CV: 27.5%). The main characteristics of lung disease are summarized in Table 1. The prevalence of DM was not uniformly distributed among lung disease categories, with a higher prevalence in cystic fibrosis patients (*p* = 0.005). Additionally, during follow-up, patients exhibited superimposed infections (87.5%) and vascular thrombosis (31.3%). Other data, such as body mass index or hypertension/diabetes mellitus status previous to LTx, could not be analyzed due to a lack of availability in data collection, which could have affected CKD progression.

The nephrology team at our SOT-related CKD clinic evaluated cases and identified the etiology of CKD as follows: CNI nephrotoxicity in 71.3% of cases (n: 57) (defined by the physician in patients with progressive renal decline without another apparent cause and no signs of glomerular damage such as proteinuria > 1.5 g/day, microhematuria, or leukocyturia), secondary thrombotic microangiopathy (TMA) in 11.3% (9 cases), tubulointerstitial disease in 8.8% (7 cases), vascular disease in 6.3% (5 cases), and glomerulopathies in 2.5% (2 cases). No tubular–interstitial lesion markers were measured in non-biopsied patients not available in our clinical practice, potentially leading to the misclassification of the same patients as CNI nephrotoxicity.

Kidney biopsy was performed in only 17 patients (21.3%), resulting in specific diagnoses: 3 glomerulopathies (1 extra-capillary glomerulonephritis, 1 amyloidosis A, and 1 membranoproliferative glomerulonephritis; none were related to associated alveolar hemorrhage) (17.7%), 2 cases of TMA (11.8%), 10 cases of CNI toxicity (58.8%), and 2 cases of tubulointerstitial nephritis (11.8%) (Table 2). In 41.3% of patients, an alternative diagnosis to CNI toxicity was established. Notably, patients who underwent biopsy had a higher degree of albuminuria compared to the non-biopsy group (a classic predictor of kidney disease progression) and required RRT earlier (15 vs. 10 years). Cystic fibrosis patients exhibited a higher percentage (52.4%) of A3 KDIGO stages compared to other groups (Table 1).

**Figure 2:**
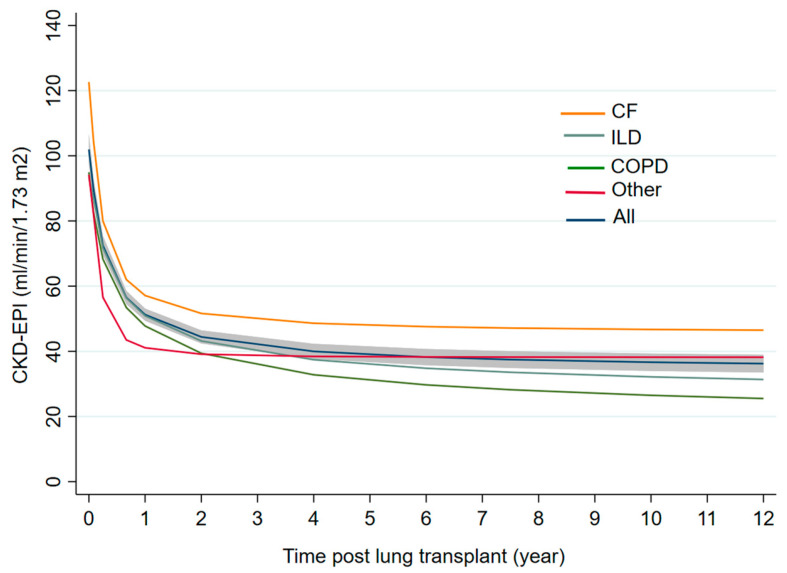
Estimated glomerular filtration rate (eGFR) by CKD-EPI formula during 12 years after lung transplant. COPD: chronic obstructive pulmonary disease, CF: cystic fibrosis, ILD: interstitial lung disease; CKD-EPI: Chronic Kidney Disease Epidemiology Collaboration.

CKD progression: primary eGFR loss occurred from transplantation surgery to the third post-LTx month, reflecting acute kidney injury (AKI) without complete recovery. This AKI resulted in a 50% decline from their pre-surgery eGFR by the first year, with no significant differences observed among different lung disease diagnoses. Subsequently, in the second phase, CKD progressed at a slower but steady rate, with an additional loss of 9.9% of eGFR by the second year (Figure 2). Other clinical events, such as infections, pulmonary rejection episodes, AKI occurring after the first year of LTx, or immunosuppressive therapy type, were not associated with CKD progression.

Nearly half of the patients experienced a MARE within the first year after LTx, with 47.4% experiencing doubling of serum creatinine, a highly significant finding. Risk factors associated with this occurrence were bipulmonary LTx (OR 8.1 [1.7–39.0]) and cystic fibrosis disease (OR 3.7 [1.1–12.3]), using interstitial lung disease as the reference category.

**Figure 3:**
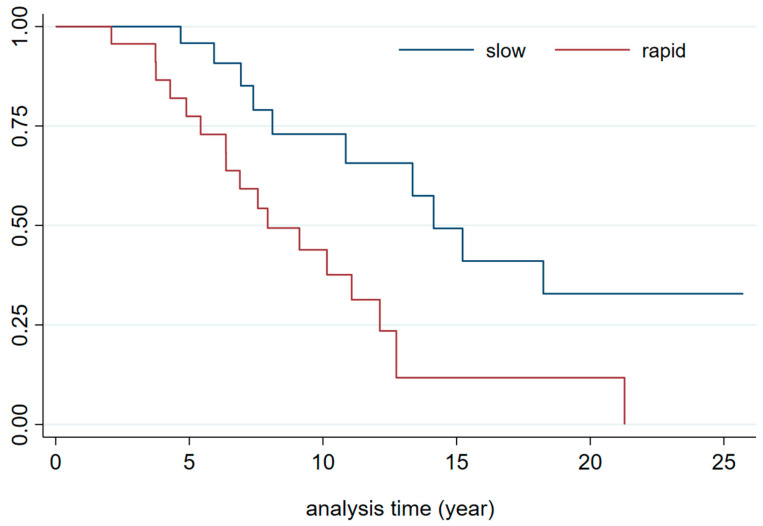
Kaplan–Meier curve until MARE/Death/RRT by kidney function loss profile. MARE: major adverse renal event; RRT: renal replacement therapy.

First-year CKD progression: Patients were categorized as slow progressors (up to 40% loss), medium progressors, and rapid progressors (greater than 60% loss). As expected, rapid progressors required RRT earlier compared to slow progressors (HR 3.4 [1.6–7.4], *p =* 0.002, Figure 3), and they were also referred to the nephrologist sooner (5.5 vs. 7.7 years [mean], *p =* 0.1).

A higher incidence of rapid progressors was observed in the cystic fibrosis group (40%) but not in other lung disease categories. The progression of kidney function, whether rapid or slow, depending on the pulmonary etiology, appears to be similar. Slow progressors demonstrated a higher percentage of male patients (72% vs. 48%; *p =* 0.2) and a higher prevalence of DM (64 vs. 48%, *p =* 0.25), with no other notable differences in terms of age or the presence of hypertension or CV disease.

Renal replacement therapy (RRT): Out of the 80 patients, 35 (43.8%) required RRT, while 8 patients died before initiating dialysis. Only 36.8% of patients with renal etiology due to CNI required dialysis compared with 60.9% (*p =* 0.05) in the other etiologies. The mean time from LTx until RRT was 12.7 years [8.1–17.0], as determined by Kaplan-Meier analysis, censored by death, with no differences between those with a renal etiology associated with CNI and the rest (12.7 [6.3–17.0] vs. 12.1 [8.1–18.2]; *p* value *=* 0.5. 

Patients with COPD exhibited a faster CKD progression and required RRT approximately 7.8 years after LTx (SD 0.8), with half of the group being on RRT at the time of the study. Cystic fibrosis patients had a mean survival time without RRT need of 12 years (SD 0.7), and those with interstitial lung disease had a mean survival time without RRT need of 13 years (SD 1.7), although this difference did not reach statistical significance (*p =* 0.2).

Given that death is considered a competitive event (Pepe Mori test *p*-value < 0.05), a competing risk test was conducted to evaluate risk factors for RRT need. In the competitive event survival analysis, lung disease and first-year CKD progression were associated with the need for RRT. First-year rapid progressors had a four times higher risk of RRT corrected by the main lung disease compared to slow progressors (see Table 3 and Figure 3). Thus, events occurring in the first year after LTx significantly impact early admission to dialysis programs. Neither albuminuria nor demographic factors at the time of referral to the nephrology clinic (such as DM or previous CV events) were associated with a higher risk of RRT need.

At the end of the follow-up, the mortality rate was 43.8% among dialysis patients compared to 17.8% among those who remained under nephrology consultation. This difference was statistically significant (*p* = 0.001), although patients on dialysis generally had a longer follow-up time.

## 4. Discussion

To our knowledge, this is the first study conducted in an outpatient clinic specifically dedicated to CKD in SOT recipients, offering a comprehensive analysis with long-term follow-up from LTx to the need for RRT while examining associated risk factors. This single-center study was conducted at one of the seven national reference centers for LTx in Spain, featuring detailed follow-up and no exclusion criteria, dropouts, or missing data. We have identified the likely causes of CKD, delineated outcomes, and explored risk factors, as well as potential areas for improvement in the management of LTx recipients.

We observed that within a few months after LTx, patients experienced a significant decline in their eGFR, losing about half of their pre-surgery eGFR. As we will describe later on, this event is crucial in CKD progression and in the complications that LTx may develop over time.

CKD in LTx patients has emerged as a significant concern in clinical practice due to the increased survival of recipients [2,3,4,11,12]. However, there is a paucity of data regarding CKD incidence in LTx recipients and the strategies for its prevention. Furthermore, the lack of an unbiased measure of renal function in these patients may lead to an underestimation of the CKD severity [13,14,15]. Consequently, a highly variable CKD incidence in LTx recipients has been reported, ranging from 16% to 70% at 5 years post-transplant [2,4,16]. For instance, Solé et al. [2] described a prevalence of CKD (defined as eGFR below 60 mL/min) at 5 years of LTx ranging from 59% to 69% in a Spanish multicentric observational study.

Previous studies have identified various risk factors associated with greater CKD progression in LTx recipients, including AKI in the immediate post-LTx period, the use of cardiopulmonary bypass, a low body mass index, underlying lung disease, and the type and dosage of immunosuppression drugs, particularly CNI [12,14,15,16,17,18,19,20,21,22,23,24,25,26]. Minimizing CNI doses or combining them with mammalian target of rapamycin (mTOR) inhibitors, when feasible, has been recognized as a valuable nephroprotective strategy, provided that the immunosuppressive efficacy is maintained [27,28,29,30]. Although previous studies conducted at our hospital identified genetic factors associated with CNI susceptibility, we were unable to replicate these analyses in our LTx patients [31].

In our sample, a significant eGFR loss was observed quite early, within the first three months after LTx, likely attributable to a multifactorial post-surgical AKI that was not fully resolved. Consequently, nearly half of the patients experienced MARE by doubling serum creatinine within the first year. Risk factors associated with CKD progression during this initial period included receiving a bipulmonary transplant and having cystic fibrosis as the underlying diagnosis. This last factor may be related to body mass index and pre-LTx overestimation of eGFR using conventional formulas that are not tailored to malnourished patients.

Our LTx patients also present other modifiable risk factors [8] that nephrologists can assist with in order to improve CKD prognosis, including albuminuria, anemia, metabolic acidosis, bone mineral disease, or CV risk. Notably, in our study LTx patients exhibited a high burden of vascular damage at the time of their first SOT-related CKD consultation (80% hypertension, 53% DM, and 27.5% with established CV disease). While the Heart and Lung Society and KDIGO guidelines lack specific recommendations for the diagnosis and treatment of CKD in LTx recipients [9,32], we recommend at least applying KDIGO guidelines for general CKD management and tailoring interventions according to CKD stage and risk of progression [8].

Although KDIGO guidelines identify albuminuria as a powerful predictor for CKD progression, our study did not find albuminuria or demographic factors at the time of referral to the nephrologist (such as DM or previous CV events) to be associated with a higher risk of initiating dialysis. It is important to note that although albuminuria was specifically measured at the first visit to the SOT-CKD clinic, it was not protocolized in the standard pulmonologist follow-up. Therefore, regular monitoring of renal function and proteinuria in isolated urine samples should be protocolized to establish the CKD diagnosis, staging, and risk of progression in all LTx recipients.

As anticipated, patients categorized as rapid progressors within the first year required RRT earlier than slow progressors. Therefore, identifying rapid progressors within the first year post-LTx is crucial for prompt nephrology referral, given the associated risks. Beyond this early phase, CKD progression occurred at a more gradual rate, with an average decline in eGFR of 12.7 mL/min per year, which remains a significant CKD progression rate. A relevant detail about our sample is the potential selection bias toward more severe patients, given the way in which they are included. 

The late referral to our clinic, more than 4 years after LTx with an eGFR of 32 mL/min, explains a dialysis-free time of less than two years for those who required it after the first nephrologist consultation. This suggests that patients were evaluated for the first time in an advanced CKD stage (grade IIIB-IV), too late to obtain a significant benefit from nephrological intervention. Our findings underline the importance of raising awareness within the SOT community about early CKD diagnosis and treatment, including the critical period immediately after transplant surgery, promoting etiological diagnosis assessment, and implementing nephroprotective measures.

Furthermore, it is essential to identify different mechanisms of kidney injury beyond CNI toxicity, as these may benefit from specific treatments to reverse kidney damage or slow CKD progression. As previously mentioned, 41.6% of patients in our study underwent an alternative CKD diagnosis after nephrological evaluation, including conditions such as TMA [33], tubulointerstitial disease, and specific vascular or glomerular diseases. Therefore, it is crucial to maintain a broad differential diagnosis, consider kidney biopsy when necessary, and avoid assuming CNI toxicity as the sole cause of kidney failure in SOT patients.

As previously reported, RRT has become a new reality in the follow-up of LTx patients, with some of them ultimately requiring kidney transplantation [34]. In our series, 43.8% of patients eventually required RRT, which was associated with a higher mortality rate (*p* < 0.001), although observed after a longer follow-up period. The majority of patients underwent RRT via hemodialysis, while only a few opted for peritoneal dialysis, which remained a feasible option alongside kidney transplantation [16,35,36,37,38,39,40,41]. We strongly advocate for the evaluation and follow-up of these patients at experienced centers with multidisciplinary teams comprising pulmonologists, nephrologists, renal nurses, and nutritionists.

Study limitations: This is a retrospective observational study, allowing only associations between variables without establishing causality. The study was conducted at a single reference center and included only patients referred to the SOT-CKD clinic, most of whom had advanced CKD (with a median referral time of 4.7 years post-LTx). This may underestimate mild CKD cases, overestimate disease progression, and limit generalizability to all LTx patients. Additionally, multiple medication changes throughout follow-up and the great variability of CNI levels, depending on clinical situations, limit the assessment of CNI’s role in CKD progression.

## 5. Conclusions

We recommend raising awareness among LTx specialists about monitoring kidney function and proteinuria, especially during the first post-transplant months. Early kidney damage predicts the need for timely nephrology evaluation, enabling protective measures and accurate diagnosis. In the absence of specific LTx guidelines, KDIGO CKD recommendations should be applied with added cardiovascular protection. These findings support future discussions on specialized care models and the need for further validation studies.

## Figures and Tables

**Table 1:** Demographic, clinical, and laboratory data.

	Total	COPD	CF	ILD	Others	*p* Value
N	80	27	23	26	4	
Previous Lung Tx						
Age (years)	49.7 (15.9)	59.9 (5.1)	28.0 (10.9)	56.9 (7.8)	54.5 (9.3)	<0.001 ^b^
Male (%)	56.3	66.7	47.8	57.7	25	0.3 ^c^
Former smokers	60.8	100	4.4	0.68	75	<0.001 ^c^
Lung transplant						
Bipulmonary (%)	81	77.8	95.7	65.4	100	0.02 ^c^
Lung disease (%)	-	33.8	28.8	28.8	5	-
Retransplant (n)	5	0	4	1	0	-
eGFR (mL/min/1.72 m^2^)	101.6 (20.1)	94.7 (17.1)	122.4 (17.5)	93.3 (13.8)	94.1 (6.1)	<0.001 ^b^
Referral to Nephrology						
eGFR (mL/min/1.72 m^2^)	31.7 (15.5)	32.6 (16)	33.2 (16.5)	30.5 (15.6)	23.9 (4.7)	0.7 ^b^
Time since LTx	4.7 [2.7–8.2]	3.1 [1.9–4.8]	9.0 [5.7–13.7]	4.7 [1.8–6.5]	12.7 [3.2–19.5]	<0.001 ^a^
Comorbidities (%)						
High blood pressure	80	85.2	78.3	73.1	100	0.5 ^c^
Diabetes mellitus	53.8	40.7	82.6	38.5	75	0.005 ^c^
Dyslipidemia	46.3	55.6	34.8	42.3	75	0.3 ^c^
Previous cardiovascular events	27.5	22.2	17.4	42.3	25	0.2 ^c^
UACR at consultation (mg/g)	41.7 [8.1–249.7]	88 [11.4–243]	44.8 [6.5–559]	40.6 [2.7–114]	14.9 [0–376]	0.7 ^a^
UACR stage (%)						0.2 ^c^
A1	32.0	30.8	19.1	37.5	75.0	
A2	36.0	46.2	28.6	37.5	0	
A3	32.0	23.1	52.4	25.0	25.0	
Leukocyturia (%)	14.7	7.7	23.8	12.5	25.0	0.4 ^c^
Microhematuria (%)	26.7	19.2	42.9	25.0	0	0.2 ^c^
KF loss 1st year (%)	−50.1 [36.3–59.2]	−52.6 [37.0–59.08]	−56.5 [37.2–64.4]	−48.9 [36.3–55.8]	−49.0 [0.1–65.1]	0.57 ^a^
KF loss 2nd year (%)	−9.9 [12.2 to−25.5]	−9.6 [12.2 to −25.4]	−0.7 [16.7 to −21.2]	−14.4 [8.8 to −27.1]	−25.1 [−57.1 to −4.6]	0.4 ^a^
Time since LTx until RRT (years)	12.7 [8.1–17.0]	7.8	12	13	21.3	0.2 ^d^
Clinical Outcomes						
Dialysis (%)	43.8	44.4	56.5	34.6	25	0.44 ^c^
Death (%)	32.5	33.3	21.7	38.5	50	0.53 ^c^
Dialysis or death (%)	53.8	55.6	56.5	50	50	0.96 ^c^

Kidney function estimated by formula CKD-EPI (mL/min/1.73 m^2^); COPD: chronic obstructive pulmonary disease; ILD: diffuse interstitial lung disease; CF: cystic fibrosis; eGFR: estimated glomerular filtrate rate: LTx: lung transplant; UACR: urine albuminuria–creatinine ratio; KF: kidney function; RRT: renal replacement therapy. ^a^: Kruskal–Wallis; ^b^: ANOVA; ^c^: Chi^2^; ^d^: estimated by Kaplan–Meier.

**Table 2:** Clinical and laboratory data according to available biopsy.

	Total	Non-Biopsy	Biopsy	*p* Value
N	80	63	17	
Previous LTx				
High blood pressure (%)	80	82.5	70.6	0.3
Diabetes mellitus (%)	53.8	50.8	64.7	0.3
eGFR (mL/min/1.72 m^2^)	101.6 (20.1)	101.8 (30.0)	99.1 (27.3)	0.8
Referral to Nephrology				
eGFR (mL/min/1.72 m^2^)	31.7 (15.5)	32.2 (14.5)	29.8 (19.5)	0.6
Time since LTx	4.7 [2.7–8.2]			
UACR at consultation (mg/g)	41.7 [8.1–249.7]	25 [6.5–199]	207.5 [41.25–541.5]	0.07
UACR stage (%)				0.006
A1	32.0	39.0	6.3	
A2	36.0	37.3	31.3	
A3	32.0	23.7	62.5	
Time since LTx until RRT (years)	12.7 [8.1–17.0]	15.2 [8.1–21.3]	10.1 [5.4–12.7]	0.03

LTx: lung transplant, kidney function estimated by formula CKD-EPI (mL/min/1.73 m^2^); UACR: urine–albuminuria–creatinine ratio; A: albuminuria; RRT: renal replacement therapy.

**Table 3:** Risk factors associated with renal replacement therapy with a competitive event model.

	UNIVARIATE				MULTIVARIATE			
	sHR	*p* Value	CI 95%		sHR	*p* Value	CI 95%	
Demographics								
CV	1.24	0.57	0.60	2.58				
DM	1.22	0.58	0.61	2.44				
Female	1.12	0.74	0.59	2.12				
Kidney disease								
Other	1.00							
CNI toxicity	0.74	0.371	0.38	1.44				
Lung disease								
COPD	1.00				1.00			
Cystic fibrosis	1.51	0.31	0.69	3.32	1.56	0.27	0.70	3.46
ILD	1.68	0.25	0.70	4.06	1.53	0.35	0.63	3.74
Others	4.26	0.047	1.02	17.77	7.73	<0.001	3.27	18.25
CKD progression								
Slow progressors	1.00				1.0			
medium	1.22	0.66	0.49	3.05	1.21	0.70	0.46	3.20
Rapid progressors	3.40	0.002	1.57	7.39	4.29	0.001	1.89	9.75
Albuminuria								
A1 (<30 mg/g)	1.00							
A2 (30–300 mg/g)	1.97		0.6	6.45				
A3 (>300 mg/g)	2.29		0.737	7.084				

sHR: subhazard ratio; CV: cardiovascular; DM: diabetes mellitus; CNI: calcineurin inhibitor; COPD: chronic obstructive pulmonary disease; ILD: interstitial lung disease; CI: confidence interval.

## Data Availability

The data presented in this study are available on request from the corresponding author. The data are not publicly available, as they include sensitive clinical information.

## Abbreviations

The following abbreviations are used in this manuscript:

AKI	acute kidney injury
CF	cystic fibrosis
COPD	chronic obstructive pulmonary disease
ILD	interstitial lung disease
LTx	lung transplant
MARE	major adverse renal events
SOT	solid organ transplants

## References

[1] Organización Nacional de Trasplantes Memoria de Actividad de Donación y Trasplante Pulmonar España 2022. https://www.ont.es/wp-content/uploads/2023/06/DONACION-Y-TRASPLANTE-GENERAL-2022.pdf.

[2] Solé A., Zurbano F., Borro J.M., Monforte V., Ussetti P., Santos F. (2015). Prevalence and Diagnosis of Chronic Kidney Disease in Maintenance Lung Transplant Patients: ICEBERG Study. Transpl. Proc..

[3] De La Morena M.P., Bravos M.D.L.T., Prado R.F., Delgado Roel M., García Salcedo J.A., Fieira Costa E., Rivas D.G., Maté J.B. (2010). Chronic kidney disease after lung transplantation: Incidence, risk factors, and treatment. Transpl. Proc..

[4] Ojo A.O., Held P.J., Port F.K., Wolfe R.A., Leichtman A.B., Young E.W., Arndorfer J., Christensen L., Merion R.M. (2003). Chronic Renal Failure after Transplantation of a Nonrenal Organ. N. Engl. J. Med..

[5] Bloom R.D., Reese P.P. (2007). Chronic kidney disease after nonrenal solid-organ transplantation. J. Am. Soc. Nephrol..

[6] Lamas S. (2005). Cellular mechanisms of vascular injury mediated by calcineurin inhibitors. Kidney Int..

[7] Remuzzi G., Bertani T. (1989). Renal vascular and thrombotic effects of cyclosporine. Am. J. Kidney Dis..

[8] Kidney Disease: Improving Global Outcomes (KDIGO) CKD Work Group (2024). KDIGO 2024 Clinical Practice Guideline for the Evaluation and Management of Chronic Kidney Disease. Kidney Int..

[9] Nelson J., Alvey N., Bowman L., Schulte J., Segovia M.C., McDermott J., Te H.S., Kapila N., Levine D.J., Gottlieb R.L. (2022). Consensus recommendations for use of maintenance immunosuppression in solid organ transplantation: Endorsed by the American College of Clinical Pharmacy, American Society of Transplantation, and the International Society for Heart and Lung Transplantation. Pharmacotherapy.

[10] Prischl F.C., Rossing P., Bakris G., Mayer G., Wanner C. (2021). Major adverse renal events (MARE): A proposal to unify renal endpoints. Nephrol. Dial. Transpl..

[11] Pham P.T.T., Slavov C., Pham P.C.T. (2009). Acute Kidney Injury After Liver, Heart, and Lung Transplants: Dialysis Modality, Predictors of Renal Function Recovery, and Impact on Survival. Adv. Chronic Kidney Dis..

[12] Canales M., Youssef P., Spong R., Ishani A., Savik K., Hertz M., Ibrahim H.N. (2006). Predictors of chronic kidney disease in long-term survivors of lung and heart-lung transplantation. Am. J. Transpl..

[13] Osho A.A., Castleberry A.W., Snyder L.D., Palmer S.M., Stafford-Smith M., Lin S.S., Davis R.D., Hartwig M.G. (2014). The Chronic Kidney Disease Epidemiology Collaboration (CKDEPI) equation best characterizes kidney function in patients being considered for lung transplantation. J. Heart Lung Transpl..

[14] Carillo C., Pecoraro Y., Anile M., Mantovani S., Oliva A., D’Abramo A., Amore D., Pagini A., De Giacomo T., Pugliese F. (2017). Evaluation of Renal Function in Patients Undergoing Lung Transplantation. Transpl. Proc..

[15] Husain-Syed F., Ferrari F., Birk H.W., Weimer R., Ronco C., Poll K., Hecker M., Walmrath H.-D., Seeger W., Kuhnert S. (2020). Pre-transplant renal functional reserve and renal function after lung transplantation. J. Heart Lung Transpl..

[16] Weber N.T., Bonani M., Benden C., Schleich A., Fehr T., Mueller T.F., Schuurmans M.M. (2018). Evolution of lung and kidney allograft function in patients receiving kidney after lung transplantation. Clin. Transpl..

[17] Wehbe E., Brock R., Budev M., Xu M., Demirjian S., Schreiber M.J., Stephany B. (2012). Short-term and long-term outcomes of acute kidney injury after lung transplantation. J. Heart Lung Transpl..

[18] Sikma M.A., Hunault C.C., van de Graaf E.A., Verhaar M.C., Kesecioglu J., de Lange D.W., Meulenbelt J. (2017). High tacrolimus blood concentrations early after lung transplantation and the risk of kidney injury. Eur. J. Clin. Pharmacol..

[19] Ishikawa S., Griesdale D.E.G., Lohser J. (2014). Acute kidney injury within 72 hours after lung transplantation: Incidence and perioperative risk factors. J. Cardiothorac. Vasc. Anesth..

[20] Hennessy S.A., Gillen J.R., Hranjec T., Kozower B.D., Jones D.R., Kron I.L., Lau C.L. (2013). Influence of hemodialysis on clinical outcomes after lung transplantation. J. Surg. Res..

[21] Banga A., Mohanka M., Mullins J., Bollineni S., Kaza V., Tanriover B., Torres F. (2017). Characteristics and outcomes among patients with need for early dialysis after lung transplantation surgery. Clin. Transpl..

[22] Sang L., Chen S., Nong L., Xu Y., Liang W., Zheng H., Zhou L., Sun H., He J., Liu X. (2021). The Prevalence, Risk Factors, and Prognosis of Acute Kidney Injury After Lung Transplantation: A Single-Center Cohort Study in China. Transpl. Proc..

[23] Atchade E., Barour S., Tran-Dinh A., Jean-Baptiste S., Tanaka S., Tashk P., Snauwaert A., Lortat-Jacob B., Mourin G., Mordant P. (2020). Acute Kidney Injury After Lung Transplantation: Perioperative Risk Factors and Outcome. Transpl. Proc..

[24] Jing L., Chen W., Zhao L., Guo L., Liang C., Chen J., Wang C. (2022). Acute kidney injury following adult lung transplantation. Chin. Med. J..

[25] Du W.W., Wang X.X., Zhang D., Chen W.Q., Zhang X.L., Li P.M. (2021). Retrospective analysis on incidence and risk factors of early onset acute kidney injury after lung transplantation and its association with mortality. Ren. Fail..

[26] Shashaty M.G.S., Forker C.M., Miano T.A., Wu Q., Yang W., Oyster M.L., Porteous M.K., Cantu E.E., Diamond J.M., Christie J.D. (2019). The association of post–lung transplant acute kidney injury with mortality is independent of primary graft dysfunction: A cohort study. Clin. Transpl..

[27] Stephany B.R., Boumitri M., Budev M., Alao B., Poggio E.D. (2009). Absence of Proteinuria Predicts Improvement in Renal Function After Conversion to Sirolimus-based Immunosuppressive Regimens in Lung Transplant Survivors with Chronic Kidney Disease. J. Heart Lung Transpl..

[28] Cassuto J.R., Levine M.H., Reese P.P., Bloom R.D., Goral S., Naji A., Abt P.L. (2012). The Influence of Induction Therapy for Kidney Transplantation after a Non-Renal Transplant. Clin. J. Am. Soc. Nephrol..

[29] Högerle B.A., Kohli N., Habibi-Parker K., Lyster H., Reed A., Carby M., Zeriouh M., Weymann A., Simon A.R., Sabashnikov A. (2016). Challenging immunosuppression treatment in lung transplant recipients with kidney failure. Transpl. Immunol..

[30] Miano T.A., Flesch J.D., Feng R., Forker C.M., Brown M., Oyster M., Kalman L., Rushefski M., Cantu E., Porteus M. (2020). Early Tacrolimus Concentrations After Lung Transplant Are Predicted by Combined Clinical and Genetic Factors and Associated with Acute Kidney Injury. Clin. Pharmacol. Ther..

[31] López-Ibor J.V., Citores M.J., Portoles J., Gomez-Bueno M., Sanchez Sobrino B., Muñoz A., Cuervas-Mons V., Segovia-Cubero J. (2022). Role of TGF-β1 +869T>C polymorphism in renal dysfunction one year after heart transplantation. J. Heart Lung Transpl..

[32] Bloom R.D., Doyle A.M. (2006). Kidney disease after heart and lung transplantation. Am. J. Transpl..

[33] Portoles J., Huerta A., Arjona E., Gavela E., Agüera M., Jiménez C., Cavero T., Marrero D., de Córdoba S.R., Diekmann F. (2021). Characteristics, management and outcomes of atypical haemolytic uraemic syndrome in kidney transplant patients: A retrospective national study. Clin. Kidney J..

[34] Serrano-Salazar M., Medina-Zahonero L., Janeiro-Marín D., Contreras-Lorenzo C., Aguilar-Perez M., Sanchez-Sobrino B., López-Sánchez P., Ussetti-Gil P., Portoles-Perez J. (2019). Kidney Transplantation in Patients With Chronic Kidney Disease After a Previous Lung Transplantation. Transpl. Proc..

[35] Lonze B.E., Warren D.S., Stewart Z.A., Dagher N.N., Singer A.L., Shah A.S., Montgomery R.A., Segev D.L. (2009). Kidney transplantation in previous heart or lung recipients. Am. J. Transpl..

[36] Srinivas T.R., Stephany B.R., Budev M., Mason D.P., Starling R.C., Miller C., Goldfarb D.A., Flechner S.M., Poggio E.D., Schold J.D. (2010). An Emerging Population Kidney Transplant Candidates Who Are Placed on the Waiting List after Liver, Heart, and Lung Transplantation. Clin. J. Am. Soc. Nephrol..

[37] Cassuto J.R., Reese P.P., Sonnad S., Bloom R.D., Levine M.H., Olthoff K.M., Shaked A., Naji A., Abt P. (2010). Wait list death and survival benefit of kidney transplantation among nonrenal transplant recipients. Am. J. Transpl..

[38] Otani S., Levvey B.J., Westall G.P., Paraskeva M., Whitford H., Williams T., McGiffin D.C., Walker R., Menahem S., Snell G.I. (2015). Long-term successful outcomes from kidney transplantation after lung and heart-lung transplantation. Ann. Thorac. Surg..

[39] Buffet A., Guillouët S., Lobbedez T., Ficheux M., Lanot A., Béchade C. (2018). Safety of peritoneal dialysis after nonrenal solid-organ transplantation. Perit. Dial. Int..

[40] El-Husseini A., Aghil A., Ramirez J., Sawaya B., Rajagopalan N., Baz M., Mei X., Davenport D.L., Gedaly R. (2017). Outcome of kidney transplant in primary, repeat, and kidney-after-nonrenal solid-organ transplantation: 15-year analysis of recent UNOS database. Clin. Transpl..

[41] Osho A.A., Hirji S.A., Castleberry A.W., Mulvihill M.S., Ganapathi A.M., Speicher P.J., Yerokun B., Snyder L.D., Davis R.D., Hartwig M.G. (2017). Long-term survival following kidney transplantation in previous lung transplant recipients—An analysis of the UNOS registry. Clin. Transpl..

